# Lung Cancer Diagnosis From Computed Tomography Images Using Deep Learning Algorithms With Random Pixel Swap Data Augmentation: Algorithm Development and Validation Study

**DOI:** 10.2196/68848

**Published:** 2025-09-03

**Authors:** Ayomide Adeyemi Abe, Mpumelelo Nyathi

**Affiliations:** 1 Department of Medical Physics, Sefako Makgatho Health Science University, Molotlegi St, Zone 1, Garankuwa, Pretoria, 0208, South Africa, 27 813208348

**Keywords:** lung cancer diagnosis, deep learning, data augmentation, convolutional neural network, transformer, random pixel swap

## Abstract

**Background:**

Deep learning (DL) shows promise for automated lung cancer diagnosis, but limited clinical data can restrict performance. While data augmentation (DA) helps, existing methods struggle with chest computed tomography (CT) scans across diverse DL architectures.

**Objective:**

This study proposes Random Pixel Swap (RPS), a novel DA technique, to enhance diagnostic performance in both convolutional neural networks and transformers for lung cancer diagnosis from CT scan images.

**Methods:**

RPS generates augmented data by randomly swapping pixels within patient CT scan images. We evaluated it on ResNet, MobileNet, Vision Transformer, and Swin Transformer models, using 2 public CT datasets (Iraq-Oncology Teaching Hospital/National Center for Cancer Diseases [IQ-OTH/NCCD] dataset and chest CT scan images dataset), and measured accuracy and area under the receiver operating characteristic curve (AUROC). Statistical significance was assessed via paired *t* tests.

**Results:**

The RPS outperformed state-of-the-art DA methods (Cutout, Random Erasing, MixUp, and CutMix), achieving 97.56% accuracy and 98.61% AUROC on the IQ-OTH/NCCD dataset and 97.78% accuracy and 99.46% AUROC on the chest CT scan images dataset. While traditional augmentation approaches (flipping and rotation) remained effective, RPS complemented them, surpassing the performance findings in prior studies and demonstrating the potential of artificial intelligence for early lung cancer detection.

**Conclusions:**

The RPS technique enhances convolutional neural network and transformer models, enabling more accurate automated lung cancer detection from CT scan images.

## Introduction

### Background

Lung cancer is a lethal disease characterized by uncontrolled cell growth in the lungs [1]. These malignant cells can proliferate, invade nearby tissues, and metastasize to other parts of the body [2]. The disease progresses through distinct stages, with advanced stages often proving fatal [3]. Lung cancer comprises multiple histological types and subtypes, affecting individuals regardless of gender [4]. Globally, lung cancer remains the leading cause of cancer-related mortality [5]. In 2020 alone, it accounted for 1.8 million deaths, ranking as the 6th leading cause of death worldwide among individuals younger than 70 years [2]. A key contributor to this high mortality is the frequent absence of early symptoms, leading to late-stage diagnosis and poorer outcomes [6]. The 5-year survival rate for lung cancer patients remains low, emphasizing the critical need for early detection [7]. Early diagnosis significantly improves prognosis, reduces long-term treatment costs, expands therapeutic options, and alleviates the burden on caregivers and families [1,8-10]. However, most cases are still detected at advanced stages, drastically limiting survival rates [5]. These challenges underscore lung cancer as a major public health priority.

Computed tomography (CT) is a medical imaging technique that produces high-resolution cross-sectional images of the lungs, providing detailed anatomical information for clinical evaluation [11]. As a noninvasive diagnostic tool, CT imaging has become indispensable for the early detection of lung cancer, offering superior sensitivity compared to conventional radiography [12,13]. However, the interpretation of CT scans presents significant challenges in clinical practice. The process demands considerable expertise from radiologists, as subtle early-stage malignancies may demonstrate imaging features that escape human detection, potentially leading to diagnostic oversights [14,15]. The subjective nature of image interpretation introduces variability in diagnostic accuracy among practitioners, which can result in false-positive identification of pulmonary nodules. Such errors may prompt unnecessary invasive procedures for confirmation, exposing patients to avoidable risks and health care systems to additional costs [13]. Furthermore, the comprehensive evaluation of CT examinations is particularly demanding, as each study comprises numerous sequential slices, requiring both individual assessment and integrated analysis. This labor-intensive process frequently overwhelms available radiological resources, contributing to diagnostic delays and extended patient waiting periods [15-17]. To address these limitations, computer-assisted diagnostic systems have been developed to augment radiologists’ interpretive capabilities [18]. These automated solutions employ advanced algorithms to analyze CT images, enhancing diagnostic accuracy while improving workflow efficiency [19]. By integrating such technological advancements into clinical practice, health care providers can mitigate the current challenges associated with manual CT interpretation, ultimately improving patient outcomes through more timely and reliable diagnoses.

The application of computer algorithms for the automated early diagnosis of lung cancer from CT scan images has evolved considerably. Early approaches used radiomics and machine learning techniques, but recent advancements have established deep learning (DL) as the predominant methodology [20]. Unlike traditional methods that depend on manually engineered features, a process prone to bias and time constraints, DL employs artificial neural networks to autonomously extract sophisticated features through training [21]. Among DL architectures, both convolutional neural networks (CNNs) and Vision Transformers have demonstrated exceptional potential for the early detection of lung cancer [22]. CNNs gained prominence after 2012, while Vision Transformers emerged in 2020 [23], with both now leading innovations in automated CT scan analysis [18,19].

CNNs and transformers offer distinct advantages for medical image analysis. CNNs, with their inductive bias for spatial locality and translation invariance, benefit from a simpler, parameter-efficient architecture rooted in spatial priors, which is highly effective and easier to train on smaller datasets [24,25]. They specialize in extracting local features and understanding spatial relationships between adjacent pixels. In contrast, transformers excel at capturing long-range dependencies across the entire image [26]. Vision Transformers are particularly scalable, maintaining image resolution better than CNNs during processing [27]. Their parallel processing capability also enables faster training times compared to similarly complex CNNs [28], although they typically require larger training datasets to achieve comparable performance [29]. Recent developments have seen the rise of hybrid networks that combine CNN and transformer architectures, successfully integrating both local and global feature extraction to overcome the limitations of standalone approaches [30,31].

Despite their capabilities, DL models face significant data-related challenges. While these architectures proficiently automate nodule detection, classification, and segmentation in CT scans [32], they demand extensive training data to outperform radiologist interpretations [33]. The scarcity of annotated medical CT datasets presents a major constraint [34], as creating such datasets requires time-consuming, expert-driven image labeling [35]. Data augmentation (DA) has emerged as a crucial solution to expand dataset size and diversity [36], enhancing both the quantity and quality of available training samples [37]. However, selecting appropriate DA techniques for chest CT analysis remains challenging due to several factors, including the variable effectiveness of methods across different datasets and domains [38], potential label distortions and crucial information loss caused by certain transformations [39], and current limitations in improving performance for both CNN and transformer architectures [37,40]. To address these challenges, this study proposes the Random Pixel Swap (RPS) augmentation method, specifically designed to enhance the generalization capabilities of both architectural paradigms in lung cancer diagnosis from chest CT scan images.

### Related Work

The effectiveness of DA in training large neural networks was first conclusively demonstrated in 2012 [41], sparking the development of numerous innovative techniques [37]. These methods primarily fall into 2 categories: data synthesis and data transformation [36]. Data synthesis techniques generate novel samples that maintain statistical similarity to the original training data, while data transformation techniques create variations by modifying existing training samples. Both approaches effectively increase training dataset size, quality, and diversity, although they differ significantly in implementation. Data synthesis typically requires parameter learning, a process that can prove computationally intensive and often demands substantial training data to achieve optimal results [42]. In contrast, data transformation techniques generally avoid parameter learning and consequently require less computational resources. Traditional data transformation methods include fundamental image manipulations such as flipping, rotation, cropping, translation, and photometric adjustments (modifications to brightness, saturation, contrast, and hue) [36]. More sophisticated approaches like Cutout [43], Random Erasing [44], MixUp [45], and CutMix [46] have subsequently emerged, achieving state-of-the-art performance across various domains. These advanced techniques have been employed in lung cancer diagnosis from CT scan images [47-49].

The following section provides a comprehensive examination of the Cutout, Random Erasing, MixUp, and CutMix techniques, analyzing their limitations in medical imaging applications and contrasting them with the proposed RPS method. This comparative analysis establishes the foundation rationale for developing specialized augmentation approaches optimized for medical image analysis challenges.

#### Cutout DA Technique

The Cutout technique randomly selects square regions within images and masks their pixel values [43]. While effective for improving model robustness against occlusions in natural images, this approach presents significant limitations for medical CT scans. The method’s potential to eliminate critical diagnostic information (such as cancerous regions) may degrade performance [38]. Additionally, the masking process can inadvertently alter image labels, further limiting effectiveness [39]. Unlike Cutout, our RPS approach avoids information loss. It preserves diagnostic information by replacing masked regions with pixel values that are derived from other areas within the same CT scan while maintaining original labels.

#### Random Erasing DA Technique

Random Erasing extends Cutout’s functionality by supporting both square and rectangular masks of varying sizes [44]. This technique randomly selects image regions for erasure and replaces them with random pixel values. While the variable mask sizes increase dataset diversity compared to Cutout, the method still suffers from information loss and label alteration issues [36,40]. These limitations are particularly problematic for medical imaging, where preserving anatomical content is crucial.

#### MixUp DA Technique

MixUp generates new samples through linear interpolation of pixel values and labels from 2 distinct images [45]. This approach enhances model generalization by preventing label memorization and improving adversarial robustness. However, the technique’s potential to blur important anatomical boundaries and the requirement of careful hyperparameter tuning can create a bottleneck in medical contexts [47,48]. Furthermore, its convergence speed is often suboptimal [47]. RPS addresses these limitations by operating within single patient scans rather than mixing data across patients and employs a single hyperparameter for more efficient training.

#### CutMix DA Technique

**Figure 1.**
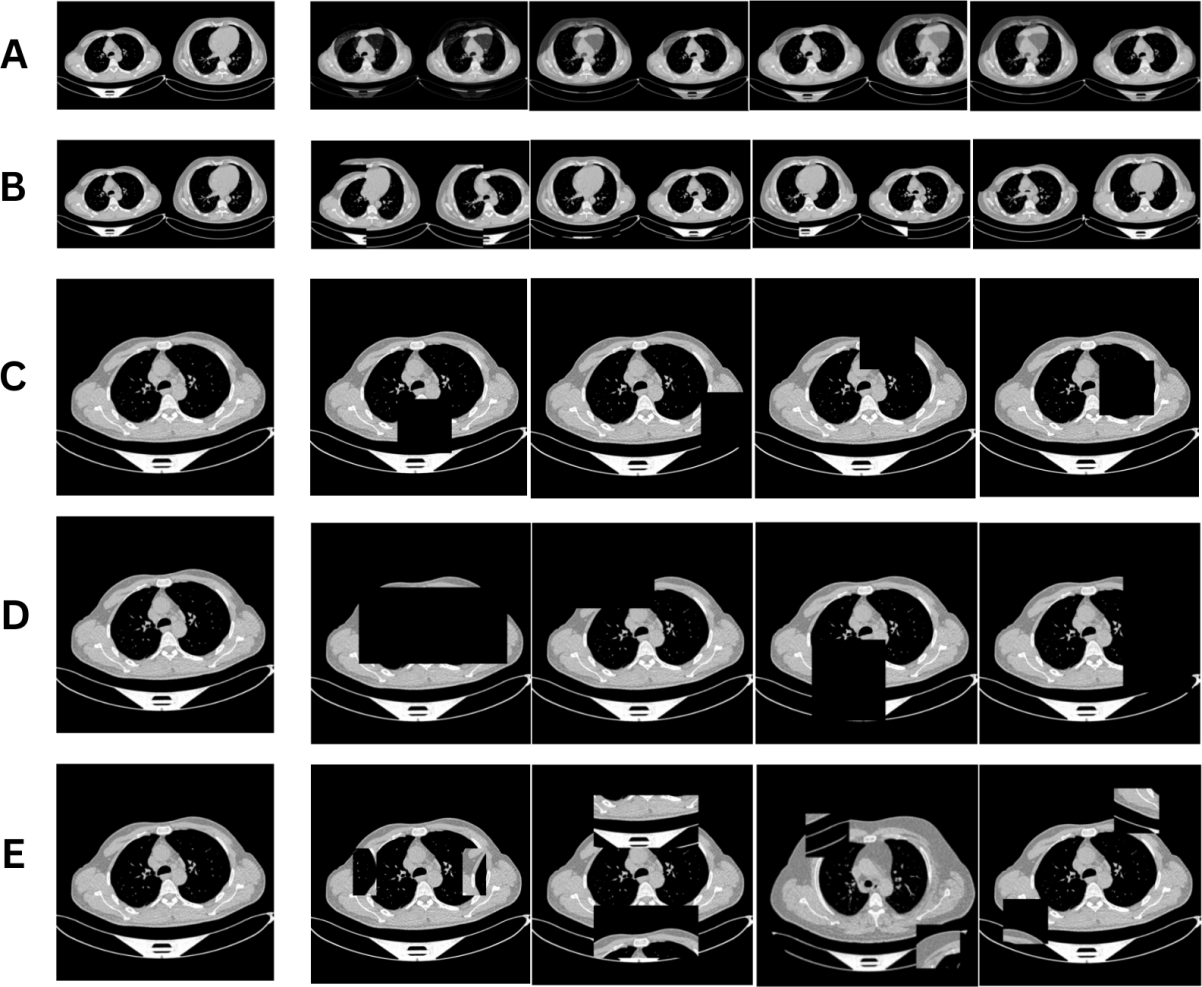
Computed tomography images for various data augmentation techniques. (A) MixUp; (B) CutMix; (C) Cutout; (D) Random Erasing; (E) Random Pixel Swap. The original image is in column 1, while the augmented images are in columns 2, 3, 4, and 5.

CutMix combines aspects of previous methods by cutting patches from one image and pasting them onto another while proportionally blending labels [46]. Although this approach leverages the benefits from both Cutout and MixUp, the label blending can introduce noise that degrades model performance [50]. For medical CT scans, combining patches from different patients may confuse learning models, particularly when dealing with subtle pathological features [51]. RPS overcomes these challenges by performing pixel swaps exclusively within individual patient scans and preserving original labels without blending. Figure 1 visually contrasts these techniques with the proposed RPS method.

## Methods

### RPS DA Technique

**Figure 2.**
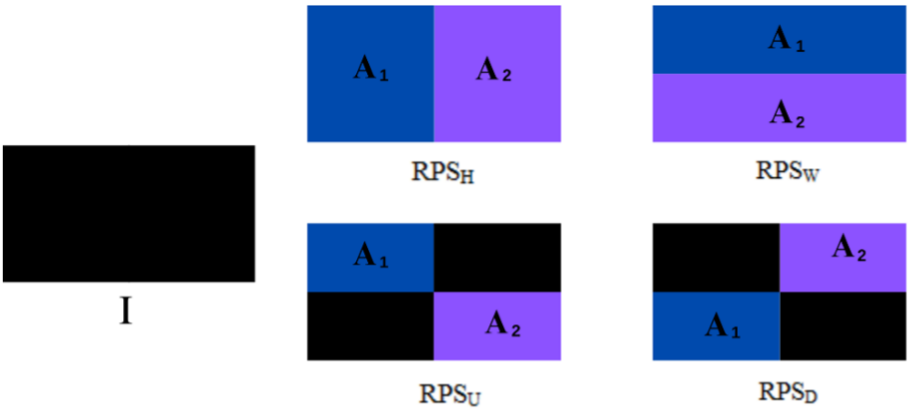
Four possible swap approaches for the Random Pixel Swap (RPS) data augmentation technique. I is the original image. Areas A_1_ and A_2_ are the swap regions. RPS_H_ (vertical), RPS_W_ (horizontal), RPS_U_ (upper right diagonal), and RPS_D_ (upper left diagonal) are the possible swap configurations.

The RPS technique is a parameter-free DA algorithm that operates with a predefined transformation probability. This method partitions input images into 2 distinct regions that serve as source and target areas for patch selection and swapping operations. The study proposes 4 specific implementation approaches, designated as RPS_H_ (vertical), RPS_W_ (horizontal), RPS_U_ (upper right diagonal), and RPS_D_ (upper left diagonal) swap configurations, as illustrated in Figure 2. This multidirectional swapping mechanism provides several advantages: it generates diverse transformations within individual patient CT scans while maintaining pathological plausibility, introduces meaningful variability in the training dataset without requiring parameter learning, and preserves all critical diagnostic information by operating exclusively within each scan’s original pixel values. The technique’s ability to produce multiple distinct transformations from a single image significantly enhances dataset diversity while avoiding the label alteration and information loss issues associated with other augmentation methods.

RPS possesses distinct invariant properties compared to other techniques. For an image with N pixels and $L_{i}$ intensity levels, the RPS transformation preserves global intensity, as shown in Equations (1)-(3). The technique employs a controlled, systematic, random patch-based pixel swap, rather than a random point-based pixel swap, ensuring that image content is preserved. This approach generates meaningful variations while maintaining pathological truth, thereby retaining clinical relevance in the context of lung cancer diagnosis.


(1) $$X^{\prime }=T(X)$$

where T is permutation transform


(2) $$p=\frac{n\left(i\right)}{N}=\frac{n{\left(i\right)}^{\prime }}{N^{\prime }};i=0,1,2,...,L-1$$

(3) $$I_{g}=\sum_{i=0}^{L-1}\frac{i}{N}=\sum_{i=0}^{L-1}\frac{i^{\prime }}{N^{\prime }}$$

where *P* is the probability of a pixel having intensity *i*; $n\left(i\right),n{\left(i\right)}^{\prime }$ is the number of pixels with intensity level $i\in X\wedge X^{\prime }$, respectively; $N,N^{\prime }$ is the total number of pixels in $X\wedge X^{\prime }$, respectively; L is the intensity level; and $I_{g}$ is the average global intensity.

### Implementation of RPS

The RPS technique is implemented by first randomly selecting 2 coordinate points (x₁, x₂) along the x-axis and 2 points (y₁, y₂) along the y-axis within the input image. These coordinates define 2 equal subswap regions: region X bounded by swap area $A_{1}$:(x₁, y₁) and (x₂, y₂), and $A_{s2}$ bounded by swap area $A_{2}$:(x₁, y₁)′ and (x₂, y₂)′. The method incorporates a key hyperparameter called the swap area factor $S_{f}$, which ranges from 0.1 to 1.0, to control the extent of augmentation. The actual swap areas $S_{a1}$ and $S_{a2}$ are derived by scaling the subswap regions using this factor, as specified in Equations(4)  (5). During the augmentation process, the contents of swap area $S_{a1}$ are cropped and pasted into swap area $S_{a2}$ while simultaneously transferring the contents of swap area $S_{a2}$ to swap area $S_{a1}$. This bidirectional swapping ensures comprehensive data transformation while preserving all original image information. The complete RPS procedure is formally described in Textbox 1.


(4) $$S_{a1}=\left(A_{s1}*S_{f}\right)$$

(5) $$S_{a2}=\left(A_{s2}*S_{f}\right)$$

Textbox 1. **Algorithm 1: Random Pixel Swap data augmentation procedure.**

**Input:** data X; with shape H×W

**Output:** Augmented data X∗

1: $A_{1}\in \frac{1}{2}(H*W)$

2: **Init:** All points P within $A_{1}$

3: $S_{f}$ ← $S_{f}$ ϵ ℚ : $S_{f}$ ϵ [0.1, 1.0]

4: **for** $P_{i},P_{j}\epsilon P,do$

5: Randomly select $P_{i}$ , $P_{j}$ ,

$P_{i}^{\prime }$ ,$P_{j}^{\prime }$ = $P_{i}*2$ , $P_{j}*2$

6: $A_{s1}$ = Area ($P_{i}$ , $P_{j}$ )

7: $A_{s2}$ = Area ($P_{i}^{\prime }$ , $P_{j}^{\prime }$ )

8: $S_{a1}$ = $A_{s1}$ * $S_{f}$

9: $S_{a2}$ = $A_{s2}$ * $S_{f}$

10: X* ←Replace $S_{a1}$ with $S_{a2}$ in X and $S_{a2}$ with $S_{a1}$ in X

11: **end for**

12: **return** X*

### Swap Area Factor

The swap area factor $S_{f}$ is a crucial parameter in the RPS technique, representing the ratio between the subswap region and the total swap area as described in Equation (6). This factor plays a vital role in the augmentation process for two key reasons: (1) it allows customization for different DL architectures that may benefit from varying swap region sizes, and (2) it helps maintain clinical relevance by limiting distortion of diagnostically important anatomical features. The study proposes two distinct implementations of this parameter: (1) single-value swap area factor (SVSF), which applies a fixed value throughout the augmentation process, and (2) multivalue swap area factor (MVSF), which uses multiple values to generate more diverse swap areas. In both implementations, the swap area factor operates within a defined range of 0.1 to 1.0, providing controlled flexibility for different medical imaging scenarios.


(6) $$S_{f}=\frac{A_{s}}{S_{a}}$$

### Experimental Validation of the RPS Technique

We conducted comprehensive experiments to validate the effectiveness of the proposed RPS technique in enhancing DL model performance across both CNN and transformer architectures. For our evaluation, we selected 4 established models: ResNet-34 [52], MobileNetV3 (small variant) [53], Vision Transformer (base-16) [23], and Swin Transformer (tiny version) [29], all initialized with preactivated weights. These architectures were chosen based on three key criteria: (1) public availability for reproducible benchmarking, (2) widespread adoption in methodological comparisons [29,48], and (3) efficient training characteristics due to their relatively fewer trainable parameters compared to larger variants.

Our experimental design incorporated three key comparisons: (1) models trained without any augmentation, (2) models trained with RPS augmentation, and (3) models trained with 4 state-of-the-art DA techniques (Cutout [43], Random Erasing [44], MixUp [45], and CutMix [46]). These comparison techniques were selected because they represent current best practices in parameter-free augmentation methods that share conceptual similarities with RPS [48]. We evaluated all models using two key metrics: (1) classification accuracy and (2) area under the receiver operating characteristic curve (AUROC), providing a comprehensive assessment of both overall performance and diagnostic discrimination capability.

### Experimental Setup and Implementation

All experiments were conducted using Python 3.12.2 (Python Software Foundation) and PyTorch 2.2.2+ cu118 (PyTorch Foundation) within Jupyter Notebook 7.0.8 (IPython Project), running on an NVIDIA Quadro RTX 3000 GPU (Nvidia Corporation). We adopted the AdamW optimizer with a cross-entropy loss function, using a batch size of 16. The StepLR scheduler was configured with a step size of 10 and a gamma value of 0.5 [52]. Models were trained for 50 epochs, as additional training resulted in overfitting and performance degradation. After evaluating various learning rates, we selected 1×10⁻⁴ as it yielded optimal results. Image normalization was applied with mean and SD values of 0.5 to enhance training stability and accelerate convergence [53].

For RPS implementation, we used a swap area factor of 1.0 with an augmentation probability of 1.0 for all experiments. CNN models processed images at 512×512 and 224×224 resolutions, while transformer architectures used 224×224 resolution due to the Vision Transformer’s input size limitations. Although the Swin Transformer supports 512×512 inputs, we maintained a consistent 224×224 resolution across all transformer experiments for fair comparison. All experiments were conducted with a random seed of 42 after verifying consistent performance patterns across 3 different seeds.

### Statistical Analysis

To evaluate our hypothesis that an effective DA technique should perform consistently across both CNN and transformer architectures, we treated each technique as an independent variable and considered model performance as the dependent variable. We used paired sample *t* tests [54] to assess significant differences between techniques, considering *P* values <.05 as statistically significant.

For comprehensive technique comparison, we implemented a ranking system based on cumulative scores C (Equations(7)  (8)), where higher scores received lower rank numbers R. This approach enabled holistic performance benchmarking across all models and architectures.


(7) $$C=\sum_{model=1}^{n}model\left(A+AUROC\right)$$

(8) $$\begin{array}{l} R^{1},R^{2},R^{3},\ldots ,R^{m+1}=C_{1},C_{2},C_{3},\ldots ,C_{m+1}\\ \vee \;C_{1}§gt;C_{2}§gt;C_{3},\ldots ,§gt;C_{m+1} \end{array}$$

where **C** is cumulative score, ***R*** is rank, **m** is the total number of data augmentation techniques, **n** is the total number of selected models, **A** is accuracy, and AUROC is the area under the receiver operating characteristic curve.

### Iraq-Oncology Teaching Hospital/National Center for Cancer Diseases Dataset

The Iraq-Oncology Teaching Hospital/National Center for Cancer Diseases (IQ-OTH/NCCD) dataset contains 1097 JPEG CT images collected from 110 patients [35]. These images were obtained using a SOMATOM Siemens scanner (Siemens Healthineers) and encompass a diverse range of demographic characteristics. The dataset is organized into 3 categories: normal scans, benign tumor scans, and malignant tumor scans. Specifically, it includes 15 cases of benign tumors, totaling 120 images; 40 cases of malignant tumors, totaling 416 images; and 55 cases of normal findings, totaling 561 images. Each image has a resolution of 512×512 pixels. We divided the images in a ratio of 7:3 for training and testing.

### Chest CT Scan Images Dataset

The chest CT scan images dataset contains 1000 lung CT scans from patients diagnosed with 3 different types of lung cancers, as well as scans from healthy individuals, all in JPG format [55]. The lung cancer types included in the dataset are adenocarcinoma, squamous cell carcinoma, and large cell carcinoma. The images are organized into training, testing, and validation sets for each lung cancer category.

### Ethical Considerations

Ethics approval was obtained from the Sefako Makgatho University Research Committee (ethics reference number: SMUREC/M/12/2022:PG).

## Results

### Average Training Time Overhead

To evaluate the computational impact of the RPS technique, we measured training duration for 4 architectures (ResNet-34, MobileNetV3 [small], Vision Transformer [base-16], and Swin Transformer [tiny]) with and without RPS implementation. The training time overhead was calculated as the difference between augmented and nonaugmented training times. Experiments were conducted on both the IQ-OTH/NCCD and chest CT scan datasets using 224×224 image resolution, with results averaged across 3 independent runs for reliability.

**Figure 3.**
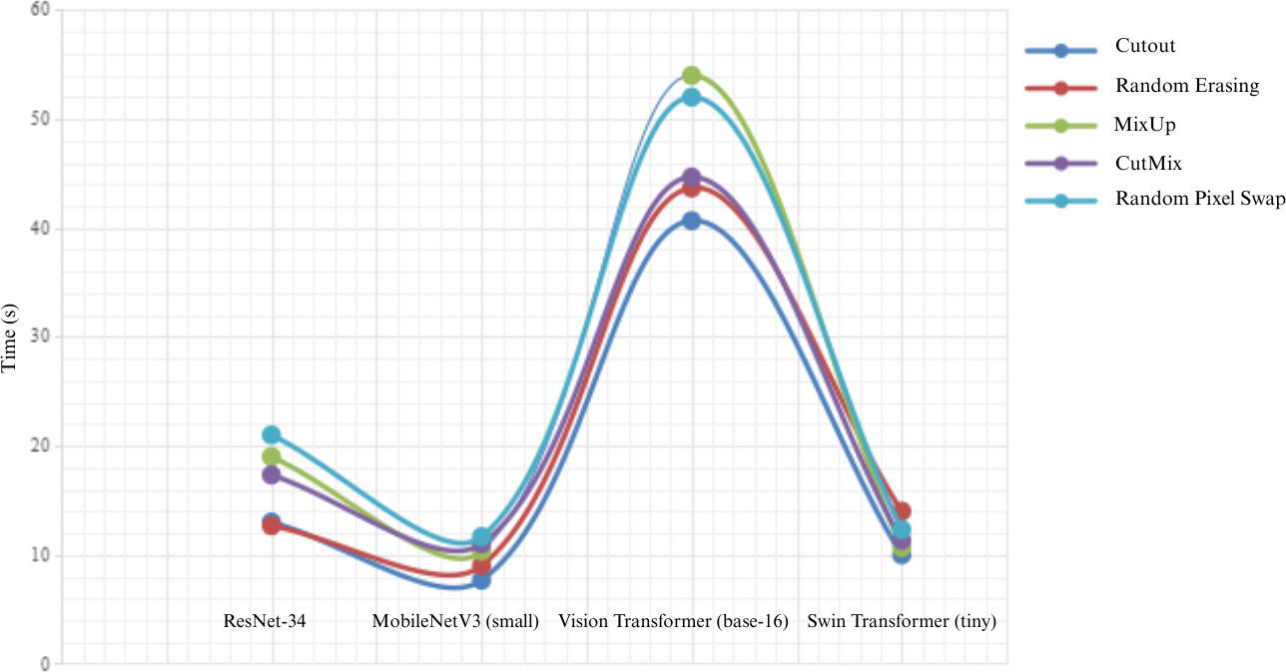
Average training time overhead of 5 data augmentation techniques across 4 deep learning models.

Our analysis included a comparative assessment of 4 established DA techniques: Cutout, Random Erasing, MixUp, and CutMix. Results demonstrated that while RPS increased training times across all models compared to nonaugmented training, this increase was not statistically significant (*P*=.07). Similarly, comparisons between RPS and other DA techniques revealed no statistically significant differences in computational overhead (Cutout: *P*=.06; Random Erasing: *P*=.17; MixUp: *P*=.49; CutMix: *P*=.16). Among all evaluated methods, RPS showed the highest training time overhead, followed sequentially by MixUp, CutMix, Random Erasing, and Cutout. Complete results are presented in Figure 3.

### Performance Comparison of RPS With State-of-the-Art DA Techniques for Lung Cancer Detection

To evaluate pulmonary nodule detection in chest CT scan images, the selected CNN and transformer models (ResNet-34, MobileNetV3 [small], Vision Transformer [base-16], and Swin Transformer [tiny]) were trained on the IQ-OTH/NCCD dataset to classify the scan images as normal or containing benign or malignant pulmonary nodules. Experimental results demonstrated that RPS significantly enhanced performance across all 4 architectures (*P*=.008). The MobileNetV3 model achieved particular success when combined with RPS using 512×512 image resolution, reaching a peak classification accuracy of 94.21%, representing a 1.22% accuracy improvement and 0.86% AUROC increase over the baseline model.

At 224×224 image resolution, our comprehensive comparison of RPS against the 4 established DA methods (Cutout: *P*=.03; Random Erasing: *P*=.008; MixUp: *P*=.02; CutMix: *P*=.02) revealed consistent superiority of the RPS technique (*P*<.05). For ResNet-34, RPS exceeded CutMix (the best alternative) by 2.44% and Random Erasing (the least effective) by 5.49% in accuracy. MobileNetV3 showed a 0.3% improvement over Cutout (best alternative) and 1.83% over MixUp (least effective) in accuracy. Transformer architectures demonstrated even more pronounced benefits: Vision Transformer with RPS outperformed Random Erasing by 1.52% and MixUp by 16.77%, while Swin Transformer showed a 1.53% improvement over MixUp and 4.57% over Cutout in accuracy. Across all architectures, performance ranking was as follows: (1) RPS (best technique), (2) Random Erasing, (3) CutMix, (4) MixUp, and (5) Cutout. The detailed results are presented in Table 1.

**Table 1.** Classification results of the IQ-OTH/NCCD^a^ dataset using preactivated deep learning models with various data augmentation techniques (224×224 image resolution).

Data augmentation	Rank^b^	ResNet-34		MobileNetV3 (small)		Vision Transformer (base-16)		Swin Transformer (tiny)	
		Accuracy, %	AUROC^c^, %	Accuracy, %	AUROC, %	Accuracy, %	AUROC, %	Accuracy, %	AUROC, %
Base model^d^	6	85.98	83.39	86.59	93.16	57.62	64.88	85.67	89.06
Cutout^d^	5	85.67	86.11	89.33	92.95	57.01	63.45	85.37	93.68
Random Erasing^d^	2	82.62	91.23	88.72	90.10	71.65	75.41^e^	86.89	91.42
MixUp^d^	4	84.45	91.05	87.80	86.57	56.40	68.55	88.41	92.51
CutMix^d^^,^^f^	3	85.67	88.34	88.41	93.02	68.90	69.68	88.41	92.05
Random Pixel Swap^f^	1	88.11^e^	93.70^e^	89.63^e^	93.80^e^	73.17^e^	74.64	89.94^e^	94.79^e^

a IQ-OTH/NCCD: Iraq-Oncology Teaching Hospital/National Center for Cancer Diseases.

b Rank represents the overall rating for each technique, with “1” indicating the best technique across all models.

c AUROC: area under the receiver operating characteristic curve.

d Significant difference between an augmentation technique and the Random Pixel Swap technique across all models.

e Highest value in the column.

f Significant difference between training using an augmentation technique and the base model across all models.

At 512×512 image resolution, ResNet-34 exhibited nuanced performance differences between augmentation techniques: while CutMix achieved a marginal 0.31% higher accuracy than RPS, RPS demonstrated significantly superior diagnostic capability with a 5.31% improvement in AUROC. Furthermore, RPS outperformed the least effective technique (Random Erasing) by 2.13% in accuracy and 3.17% in AUROC. For MobileNetV3, RPS dominated all comparative techniques in both accuracy and AUROC, except for a 1.23% AUROC advantage by CutMix. Specifically, RPS exceeded Cutout (the best alternative technique) by 0.61% and surpassed MixUp (the least effective) by 4.58% in accuracy. Across all evaluated methods, the overall performance ranking was as follows: (1) RPS (best technique), (2) Cutout, (3) CutMix, (4) MixUp, and (5) Random Erasing. The detailed results are presented in Table 2.

**Table 2.** Classification results of the IQ-OTH/NCCD^a^ dataset using preactivated deep learning models with various data augmentation techniques (512×512 image resolution).

Data augmentation	Rank^b^	ResNet-34		MobileNetV3 (small)	
		Accuracy, %	AUROC^c^, %	Accuracy, %	AUROC, %
Base model^d^	6	88.72	78.51	92.99	94.81
Cutout^d^	2	90.24	93.25	93.60	95.42
Random Erasing^d^	5	89.94	91.85	90.85	92.19
MixUp^d^	4	89.94	96.13^e^	89.63	95.18
CutMix	3	92.38^e^	89.71	92.68	96.90^e^
Random Pixel Swap	1	92.07	95.02	94.21^e^	95.67

a IQ-OTH/NCCD: Iraq-Oncology Teaching Hospital/National Center for Cancer Diseases.

b Rank represents the overall rating for each technique, with “1” indicating the best technique across all models.

c AUROC: area under the receiver operating characteristic curve.

d Significant difference between an augmentation technique and the Random Pixel Swap technique across all models.

e Highest value in the column.

### Performance Comparison of RPS With State-of-the-Art DA Techniques for Lung Cancer Classification From CT Scan Images Using DL Architectures

We evaluated the effectiveness of the RPS technique for lung cancer classification using the chest CT scan images dataset across multiple DL architectures. The experimental results demonstrated that RPS significantly enhanced classification performance for all architectures (*P*=.008). RPS combined with ResNet-34 at 512×512 image resolution achieved optimal performance, reaching 97.78% accuracy and 99.46% AUROC.

At 224×224 image resolution, RPS consistently outperformed competing techniques across most models (Cutout: *P*=.001; Random Erasing: *P*=.02; MixUp: *P*=.047; CutMix: *P*=.18). For ResNet-34, RPS exceeded CutMix (the best alternative) by 0.64% and Random Erasing (the least effective) by 5.08% in accuracy. MobileNetV3 showed even greater improvements over other methods, with RPS surpassing CutMix by 3.49% and MixUp by 9.21% in accuracy. For the implementation with Vision Transformer, RPS surpassed Random Erasing (the best alternative) by 1.91% and MixUp (the least effective) by 18.85% in accuracy. While CutMix showed a 2.22% accuracy advantage over RPS for the Swin Transformer, RPS maintained superior performance against all other techniques, exceeding Cutout by 7.3% (the least effective). Across all architectures, the overall performance ranking was as follows: (1) RPS (best technique), (2) CutMix, (3) Random Erasing, (4) Cutout, and (5) MixUp. The detailed results are presented in Table 3.

**Table 3.** Classification results of the chest CT^a^ scan images dataset using preactivated deep learning models with various data augmentation techniques (224×224 image resolution).

Data augmentation	Rank^b^	ResNet-34		MobileNetV3 (small)		Vision Transformer (base-16)		Swin Transformer (tiny)	
		Accuracy, %	AUROC^c^, %	Accuracy, %	AUROC, %	Accuracy, %	AUROC, %	Accuracy, %	AUROC, %
Base model^d^	5	93.33	99.00	87.30	97.09	82.86	95.84	84.76	96.92
Cutout^d^^,^^e^	4	93.02	98.94	85.71	97.62	80.63	94.35	84.13	96.05
Random Erasing^d^	3	90.48	98.54	88.89	97.45	84.76	96.72^f^	88.25	97.28
MixUp^d^	6	91.43	98.57	83.49	96.85	67.82	86.97	90.79	97.87
CutMix	2	94.92	98.69	89.21	97.80	76.82	92.60	93.65^f^	98.74^f^
Random Pixel Swap^e^	1	95.56^f^	99.15^f^	92.70^f^	98.02^f^	86.67^f^	96.32	91.43	98.45

a CT: computed tomography.

b Rank represents the overall rating for each technique, with “1” indicating the best technique across all models.

c AUROC: area under the receiver operating characteristic curve.

d Significant difference between an augmentation technique and the Random Pixel Swap technique across all models.

e Significant difference between training using an augmentation technique and the base model across all models.

f Highest value in the column.

At 512×512 image resolution, the RPS technique demonstrated superior performance compared to all evaluated DA methods (Cutout: *P*=.13; Random Erasing: *P*=.27; MixUp: *P*=.13; CutMix: *P*=.31). For ResNet-34, RPS matched the accuracy of the top-performing alternative (CutMix) while achieving a 0.21% improvement in AUROC. Furthermore, RPS showed significant gains over the least effective technique (MixUp), with a 7.74% accuracy performance advantage. The MobileNetV3 architecture exhibited even more pronounced benefits, where RPS outperformed CutMix (the best alternative) by 2.23% and surpassed MixUp by 4.45% in accuracy. Across all techniques, the performance ranking was as follows: (1) RPS (best technique), (2) CutMix, (3) Cutout, (4) Random Erasing, and (5) MixUp. The detailed results are presented in Table 4.

**Table 4.** Classification results of the chest CT^a^ scan images dataset using preactivated deep learning models with various data augmentation techniques (512×512 image resolution).

Data augmentation	Rank^b^	ResNet-34		MobileNetV3 (small)	
		Accuracy, %	AUROC^c^, %	Accuracy, %	AUROC, %
Base model	5	96.83	99.25	93.02	98.27
Cutout	3	96.51	99.35	94.60	98.39
Random Erasing	4	96.83	99.42	93.65	98.82^d^
MixUp	6	92.38	98.64	92.38	98.51
CutMix	2	97.78^d^	99.25	94.60	98.61
Random Pixel Swap	1	97.78^d^	99.46^d^	96.83^d^	98.75

a CT: computed tomography.

b Rank represents the overall rating for each technique, with “1” indicating the best technique across all models.

c AUROC: area under the receiver operating characteristic curve.

d Highest value in the column.

### Performance Analysis of Swap Area Factors for Lung Cancer Diagnosis

The swap area factor serves as a critical hyperparameter in RPS implementation. We systematically evaluated its influence using both SVSF and MVSF configurations across the 0.1 to 1.0 range on the IQ-OTH/NCCD dataset. MVSF provides over 100 possible combinations of lower and upper bounds (eg, 0.1‐0.5 and 0.4‐0.8); however, our experimental configurations maintained a fixed lower bound of 0.1. Experimental results revealed distinct optimal configurations for each architecture. For SVSF implementations, ResNet-34, Vision Transformer, and Swin Transformer achieved peak performance at 1.0, while MobileNetV3 performed best at 0.9. For MVSF implementations, ResNet-34 showed optimal results within 0.1‐0.9, MobileNetV3 performed best at 0.1‐0.7, Vision Transformer excelled at 0.1‐0.3, and Swin Transformer achieved peak performance at 0.1‐0.5.

Comparative analysis demonstrated that SVSF generally outperformed MVSF configurations for a fixed 0.1 lower bound across most architectures, with the notable exception of ResNet-34. For this model, MVSF (0.1‐0.9) surpassed SVSF (1.0) by 0.61% in accuracy and 1.08% in AUROC. The most effective overall configuration combined MobileNetV3 with RPS using an SVSF of 0.9, achieving 94.51% accuracy and 95.77% AUROC. The detailed results are presented in Table 5.

**Table 5.** Analysis of the IQ-OTH/NCCD^a^ dataset using different deep learning architectures and Random Pixel Swap data augmentation with single-value and multivalue swap area factors (224×224 image resolution).

Swap factor	ResNet-34		MobileNetV3 (small)		Vision Transformer (base-16)		Swin Transformer (tiny)	
	Accuracy, %	AUROC^b^, %	Accuracy, %	AUROC, %	Accuracy, %	AUROC, %	Accuracy, %	AUROC, %
Single value								
0.1	89.02	92.26	93.90	95.10	64.02	74.97^c^	86.28	92.02
0.2	91.16	94.62	92.99	95.04	60.98	63.20	83.23	93.59
0.3	90.85	93.48	92.07	95.38	64.02	67.95	89.02	91.63
0.4	89.63	92.66	92.99	95.24	69.21	72.59	84.76	92.81
0.5	90.55	90.96	92.68	95.01	68.60	72.47	87.80	84.13
0.6	90.55	94.76	92.99	95.24	69.21	72.36	84.76	89.63
0.7	91.46	95.23	92.68	95.12	67.99	66.48	83.54	90.65
0.8	89.63	92.22	93.60	95.60	67.99	69.83	89.94^c^	95.95^c^
0.9	90.85	94.42	94.51^c^	95.77^c^	71.65	72.79	83.84	90.99
1.0	92.07^c^	95.02^c^	94.21	95.67	73.17^c^	74.64	89.94^c^	94.79
Multivalue								
0.1‐0.2	90.55	93.80	93.90^c^	94.83	66.16	69.14	85.98	94.29
0.1‐0.3	89.33	90.53	93.29	95.18	72.56^c^	77.93^c^	84.76	91.84
0.1‐0.4	89.33	90.18	93.60	95.03	60.98	73.15	87.50	94.30
0.1‐0.5	91.16	95.93^c^	93.60	94.84	59.15	68.80	88.11^c^	94.94^c^
0.1‐0.6	90.55	93.26	92.38	94.60	62.20	59.86	87.20	93.45
0.1‐0.7	89.94	90.99	93.90^c^	95.33	61.28	70.73	88.11^c^	92.53
0.1‐0.8	87.80	93.13	93.29	95.00	66.77	76.81	86.28	85.93
0.1‐0.9	92.68^c^	95.29	93.60	95.29	68.29	64.98	86.59	92.69
0.1‐1.0	89.02	92.53	93.60	95.71^c^	62.80	69.05	83.54	94.35

a IQ-OTH/NCCD: Iraq-Oncology Teaching Hospital/National Center for Cancer Diseases.

b AUROC: area under the receiver operating characteristic curve.

c Highest value in the column.

Our evaluation of the chest CT scan images dataset using different swap area factor configurations revealed architecture-specific optimal settings. SVSF demonstrated superior performance at 1.0 for both ResNet-34 and MobileNetV3, while Vision Transformer achieved peak accuracy with an SVSF of 0.1. For Swin Transformer, MVSF configurations between 0.1 and 0.6 yielded optimal results. Among all tested combinations, ResNet-34 paired with RPS using an SVSF of 1.0 delivered the highest classification performance, reaching 97.78% accuracy and 99.46% AUROC. The detailed results are presented in Table 6.

**Table 6.** Analysis of the chest CT^a^ scan images dataset using different deep learning architectures and Random Pixel Swap data augmentation with single-value and multivalue swap area factors (224×224 image resolution).

Swap factor	ResNet-34		MobileNetV3 (small)		Vision Transformer (base-16)		Swin Transformer (tiny)	
	Accuracy, %	AUROC^b^, %	Accuracy, %	AUROC, %	Accuracy, %	AUROC, %	Accuracy, %	AUROC, %
Single value								
0.1	96.19	99.13	94.60	98.55	86.67^c^	96.32	94.29^c^	98.72
0.2	97.46	99.27	94.60	98.60	81.27	94.37	92.06	98.78^c^
0.3	96.19	99.22	95.24	98.61	78.73	93.98	93.65	98.65
0.4	97.46	99.20	95.24	98.65	82.54	96.10	92.06	98.46
0.5	97.14	99.41	94.92	98.74	85.08	96.31	91.43	98.38
0.6	97.14	99.30	95.56	98.79^c^	85.40	96.86^c^	91.75	97.85
0.7	97.14	99.19	95.56	98.69	83.81	95.48	93.65	98.65
0.8	97.14	99.38	95.87	98.75	81.59	95.48	91.43	97.95
0.9	96.83	99.35	95.87	98.62	81.27	94.25	88.89	97.80
1.0	97.78^c^	99.46^c^	96.83^c^	98.75	75.56	91.62	91.43	98.45
Multivalue								
0.1‐0.2	97.14	99.27	94.92	98.59	75.56	92.85	93.65	98.51
0.1‐0.3	96.51	99.30	93.97	98.62	84.13	95.86	91.43	98.20
0.1‐0.4	96.51	99.00	94.60	98.55	76.83	92.77	93.65	98.73
0.1‐0.5	97.46	99.28	94.29	98.63	80.32	94.69	92.38	98.64
0.1‐0.6	96.51	99.37	95.24	98.59	82.54	95.63	96.19^c^	98.90^c^
0.1‐0.7	97.78^c^	99.39	94.92	98.65	86.03^c^	96.84^c^	94.29	98.90^c^
0.1‐0.8	97.46	99.25	93.97	98.62	81.90	94.88	93.02	98.83
0.1‐0.9	97.48	99.32	94.92	98.70	81.90	94.99	93.65	98.86
0.1‐1.0	97.46	99.41^c^	95.87^c^	98.75^c^	82.22	95.21	93.33	98.72

a CT: computed tomography.

b AUROC: area under the receiver operating characteristic curve.

c Highest value in the column.

### RPS With Lung Region of Interest Segmentation

Prior studies have demonstrated that segmenting lung regions of interest (ROIs) can significantly improve the diagnostic performance of DL models [33,56]. To evaluate the effectiveness of the RPS technique when applied to segmented images, we conducted experiments using the selected models (ResNet-34, MobileNetV3 [small], Vision Transformer [base-16], and Swin Transformer [tiny]). Our investigation used both the IQ-OTH/NCCD dataset and chest CT scan images dataset at 224×224 resolution.

**Figure 4.**
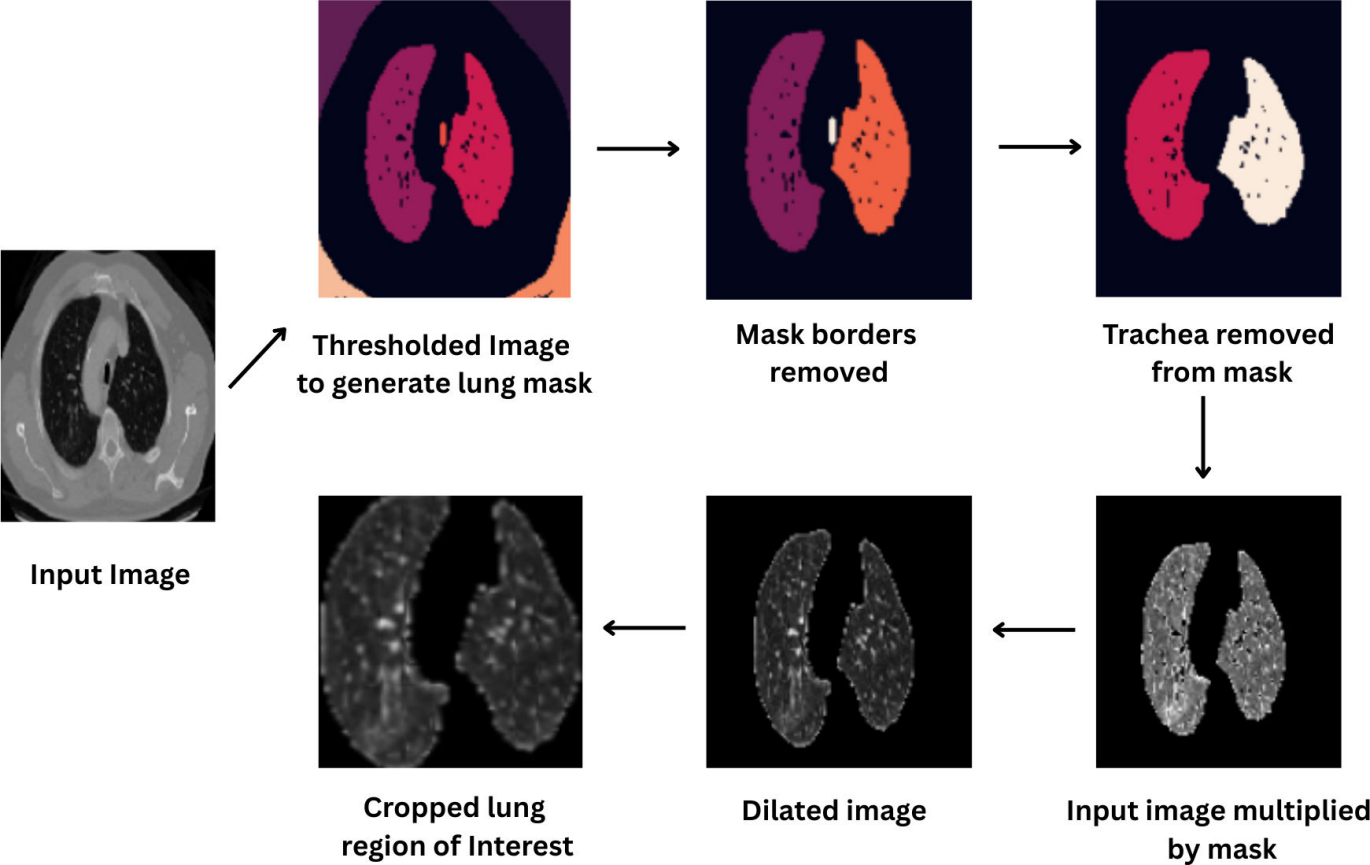
Lung segmentation procedure.

The segmentation process involved multiple steps. We first applied a threshold algorithm to generate a lung mask, followed by dilation and hole-filling operations to ensure comprehensive coverage of pulmonary structures. The final lung ROI was extracted by cropping surrounding pixels along the mask boundaries. The complete procedure is illustrated in Figure 4. For comparative analysis, we evaluated model performance under three conditions: (1) training without augmentation, (2) training with RPS, and (3) training with established augmentation techniques (Cutout, Random Erasing, MixUp, and CutMix). This comprehensive evaluation framework allowed us to assess the relative benefits of RPS when applied to segmented lung images.

Our experiments with the IQ-OTH/NCCD dataset demonstrated that the RPS technique significantly improved performance across all evaluated models (*P*=.04) and most techniques (Cutout: *P*=.049; Random Erasing: *P*=.004; MixUp: *P*=.04; CutMix: *P*=.06). The most notable results were achieved by ResNet-34 with RPS, reaching 97.56% accuracy and 98.61% AUROC. While RPS outperformed all competing techniques for MobileNetV3 and Swin Transformer, CutMix showed superior performance for Vision Transformer, exceeding RPS by 1.52% in accuracy and 0.67% in AUROC. The overall performance ranking across techniques was as follows: (1) RPS (best technique), (2) CutMix, (3) Random Erasing, (4) Cutout, and (5) MixUp.

For the chest CT scan images dataset, the RPS technique consistently improved performance across models (*P*=.06) and most techniques (Cutout: *P*=.01; Random Erasing: *P*=.009; MixUp: *P*=.01; CutMix: *P*=.38). The highest performance was again achieved by ResNet-34 with RPS (95.51% accuracy and 98.86% AUROC). While RPS showed superior results for MobileNetV3 and Swin Transformer, CutMix performed better for Vision Transformer (3.21% higher accuracy and 0.6% higher AUROC). The comprehensive performance ranking was similar to that for the IQ-OTH/NCCD dataset and was as follows: (1) RPS, (2) CutMix, (3) Cutout, (4) Random Erasing, and (5) MixUp. The detailed results are presented in Table 7.

**Table 7.** Classification results of the IQ-OTH/NCCD^a^ and chest CT^b^ scan images datasets using preactivated deep learning models with various data augmentation techniques and segmentation of the lung region of interest (224×224 image resolution).

Data augmentation	Rank^c^	ResNet-34		MobileNetV3 (small)		Vision Transformer (base-16)		Swin Transformer (tiny)	
		Accuracy, %	AUROC^d^, %	Accuracy, %	AUROC, %	Accuracy, %	AUROC, %	Accuracy, %	AUROC, %
IQ-OTH/NCCD dataset									
Base model^e^	5	96.65	99.13	95.43	97.28	89.94	96.51	93.60	98.21
Cutout^e^	4	96.04	98.86	95.43	96.33	92.38	96.20	93.90	97.80
Random Erasing^e^	3	95.73	97.45	96.65^f^	97.29	91.46	96.27	94.82^f^	98.00
MixUp^e^^,^^g^	6	95.87	99.19^f^	91.77	97.11	91.77	96.27	93.29	97.52
CutMix	2	96.65	98.86	94.51	96.39	93.90^f^	97.64^f^	93.29	97.52
Random Pixel Swap^g^	1	97.56^f^	98.61	96.65^f^	98.00^f^	92.38	96.97	94.82^f^	98.12^f^
Chest CT scan images dataset									
Base model	2	95.19	99.03	87.82	96.83^f^	82.69	95.48	90.71	98.11
Cutout^e^	4	94.55	98.85	88.14	97.66	80.77	93.86	88.14	97.32
Random Erasing^e^^,^^g^	5	94.55	98.75	86.54	96.52	79.81	89.72	86.86	97.16
MixUp^e^^,^^g^	6	94.55	98.77	82.05	95.33	78.85	93.29	85.90	97.10
CutMix	3	95.19	99.05^f^	86.54	96.89	86.86^f^	96.43^f^	87.82	96.73
Random Pixel Swap	1	95.51^f^	98.86	90.71^f^	97.51	83.65	95.83	91.35^f^	98.36^f^

a IQ-OTH/NCCD: Iraq-Oncology Teaching Hospital/National Center for Cancer Diseases.

b CT: computed tomography.

c Rank represents the overall rating for each technique, with “1” indicating the best technique across all models.

d AUROC: area under the receiver operating characteristic curve.

e Significant difference between an augmentation technique and the Random Pixel Swap technique across all models.

f Highest value in the column.

g Significant difference between training using an augmentation technique and the base model across all models.

### Performance Analysis of the Combination of RPS With Traditional DA Techniques for Lung Cancer Diagnosis

Traditionally, DA techniques, including image flipping and rotation, are widely employed in medical image analysis with DL [44]. To evaluate the potential benefits of combining these methods with the RPS technique, we conducted a systematic comparison. First, we trained selected models (ResNet-34, MobileNetV3 [small], Vision Transformer [base-16], and Swin Transformer [tiny]) using individual traditional techniques: horizontal flipping, vertical flipping, and random rotation (±90°). Subsequently, we trained the models using combinations of each traditional technique with RPS.

Our experiments revealed that the combination of RPS with traditional techniques generally enhanced model performance compared to using traditional methods alone. However, when a traditional technique failed to improve baseline performance, its combination with RPS did not surpass RPS alone. For the IQ-OTH/NCCD dataset, using RPS alone surpassed the individual traditional techniques (horizontal flipping: *P*=.63; vertical flipping: *P*=.22; rotation: *P*=.93). RPS with rotation achieved peak performance for ResNet-34 and Vision Transformer (base-16), improving upon rotation alone by 2.14% and 2.75% in accuracy, respectively. RPS with vertical flipping performed the best for MobileNetV3 (small), exceeding vertical flipping alone by 0.61% in accuracy. However, RPS alone showed superior results for Swin Transformer (tiny).

Similarly, for the chest CT scan images dataset, using RPS alone surpassed the individual traditional techniques (horizontal flipping: *P*=.01; vertical flipping: *P*=.03; rotation: *P*=.04). RPS with rotation demonstrated the strongest overall performance, improving upon rotation by 0.95% in accuracy. RPS with horizontal flipping achieved optimal results for Vision Transformer (base-16), surpassing horizontal flipping alone by 5.71% in accuracy. However, RPS alone outperformed all combinations for MobileNetV3 (small) and Swin Transformer (tiny). The detailed results are presented in Table 8.

**Table 8.** Classification results of the IQ-OTH/NCCD^a^ and chest CT^b^ scan images datasets using preactivated deep learning models when 3 traditional data augmentation techniques are combined with the Random Pixel Swap data augmentation technique (224×224 image resolution).

Data augmentation	Rank^c^	ResNet-34		MobileNetV3 (small)		Vision Transformer (base-16)		Swin Transformer (tiny)	
		Accuracy, %	AUROC^d^, %	Accuracy, %	AUROC, %	Accuracy, %	AUROC, %	Accuracy, %	AUROC, %
IQ-OTH/NCCD dataset									
Base model^e^	8	85.98	83.39	86.59	93.16	57.62	64.88	85.67	89.06
Horizontal flip	5	82.01	86.70	88.11	93.36	73.78	86.20	87.50	91.86
Horizontal flip with Random Pixel Swap	2	87.20	90.43	88.41	91.21	78.36	89.31^f^	88.72	92.12
Vertical flip^g^	7	87.50	89.82	90.55	93.89^f^	62.50	76.54	89.02	92.41
Vertical flip with Random Pixel Swap^e^^,^^g^	6	88.11	89.54	91.16^f^	93.28	68.29	73.94	88.72	93.10
Rotation^g^	4	87.80	90.30	89.63	91.53	75.91	80.70	88.41	92.67
Rotation with Random Pixel Swap^g^	1	89.94^f^	90.28	89.02	91.75	78.66^f^	87.16	89.02	93.10
Random Pixel Swap^g^	3	88.11	93.70^f^	89.63	93.80	73.17	74.64	89.94^f^	94.79^f^
Chest CT scan images dataset									
Base model^e^	8	93.33	99.00	87.30	97.09	82.86	95.84	84.76	96.92
Horizontal flip^e^	7	91.43	98.56	87.62	97.37	82.86	95.83	91.75	98.33
Horizontal flip with Random Pixel Swap^g^	4	93.97	98.96	89.21	97.48	88.57^f^	97.63^f^	92.70	98.09
Vertical flip^e^^,^^g^	6	93.33	98.86	83.81	96.83	84.72	96.10	92.06	98.58
Vertical flip with Random Pixel Swap^e^^,^^g^	5	93.97	98.81	86.67	97.13	84.76	96.23	91.43	98.04
Rotation^e^^,^^g^	3	95.24	99.22	90.16	97.58	84.57	94.95	95.87	99.03
Rotation with Random Pixel Swap^g^	1	96.19^f^	99.24^f^	91.75	97.73	85.23	95.10	96.19	98.99
Random Pixel Swap^g^	2	95.56	99.15	92.70^f^	98.02^f^	86.67	96.32	96.19^f^	98.90^f^

a IQ-OTH/NCCD: Iraq-Oncology Teaching Hospital/National Center for Cancer Diseases.

b CT: computed tomography.

c Rank represents the overall rating for each technique, with “1” indicating the best technique across all models.

d AUROC: area under the receiver operating characteristic curve.

e Significant difference between an augmentation technique and the Random Pixel Swap technique across all models.

f Highest value in the column.

g Significant difference between training using an augmentation technique and the base model across all models.

### Validation Results of the Generalization Capabilities of the RPS Technique

Enhancing the generalization ability of DL models to unseen data represents a critical objective of DA [46]. To evaluate the RPS technique’s capacity to improve model generalization, we conducted experiments using the selected models (ResNet-18, MobileNetV3 [small], Vision Transformer [base-16], and Swin Transformer [tiny]). Models were trained on the IQ-OTH/NCCD dataset and validated on the chest CT scan images dataset (distinct collections acquired using different imaging equipment, protocols, time periods, and geographical locations). All models performed binary classification (cancerous vs normal) of CT images.

Our comparative analysis included the base models, RPS implementation, and selected standard DA techniques. The results demonstrated RPS’s superior performance across all architectures (Cutout: *P*=.05; Random Erasing: *P*=.054; MixUp: *P*=.04; CutMix: *P*=.03), with an exception for the Vision Transformer implementation. Random Erasing showed a marginal 0.8% accuracy advantage over RPS. However, RPS maintained a significant 9.28% improvement in AUROC over Random Erasing. Furthermore, the cumulative ranking was as follows: (1) RPS (best technique), (2) Cutout, (3) CutMix, (4) Random Erasing, and (5) MixUp. The detailed results are presented in Table 9.

**Table 9.** Validation results of the generalization capabilities of different data augmentation techniques for lung cancer diagnosis using deep learning (224×224 image resolution).

Data augmentation	Rank^a^	ResNet-34		MobileNetV3 (small)		Vision Transformer (base-16)		Swin Transformer (tiny)	
		Accuracy, %	AUROC^b^, %	Accuracy, %	AUROC, %	Accuracy, %	AUROC, %	Accuracy, %	AUROC, %
Base model^c^	3	82.53	84.33	91.24	90.03	79.29	63.60	92.22	95.22^d^
Cutout^c^	2	82.65	97.29	92.09	90.70	81.80	63.49	92.22	85.77
Random Erasing^e^	5	88.71	78.66	91.45	89.66	82.65^d^	58.92	91.96	85.96
MixUp^c^	6	83.80	95.17	91.58	90.36	80.74	52.24	91.58	79.73
CutMix^c^	4	84.57	94.36	90.69	80.26	81.12	66.90	92.09	86.09
Random Pixel Swap^e^	1	90.69^d^	97.48^d^	92.35^d^	93.30^d^	81.85	68.20^d^	92.35^d^	95.04

a Rank represents the overall rating for each technique, with “1” indicating the best technique across all models.

b AUROC: area under the receiver operating characteristic curve.

c Significant difference between an augmentation technique and the Random Pixel Swap technique across all models.

d Highest value in the column.

e Significant difference between training using an augmentation technique and the base model across all models.

### Comparison With Prior Work

Our experimental results demonstrated improvements over the results of previous studies using both the IQ-OTH/NCCD and chest CT scan images datasets. For the IQ-OTH/NCCD dataset, our approach achieved a 7.67% performance improvement over a machine learning technique in the study by Kareem et al [57], a 4.76% improvement over an ensemble of VGG-16, ResNet-50, InceptionV3, and EfficientNetB7 models in the study by Solyman et al [58], and a 2.13% enhancement over an ensemble of 3 custom CNNs in the study by Abe et al [59]. Similarly, for the chest CT scan images dataset, our method showed a 5.78% improvement over a 3-layer custom CNN in the study by Mamun et al [60] and a 2.22% improvement over a 5-layer CNN with a custom Mavage Pooling layer in the study by Abe et al [47]. The comparative results are detailed in Table 10.

**Table 10.** Comparison of our study results with the results of previous studies on the analysis of the IQ-OTH/NCCD^a^ and chest CT^b^ scan images datasets.

Dataset and study	Accuracy, %	Number of classes
IQ-OTH/NCCD		
Kareem et al [57]	89.89	3
Solyman et al [58]	92.80	3
Abe et al [59]	95.43	3
Our study	97.56	3
Chest CT scan images		
Mamun et al [60]	92.00	4
Abe et al [47]	95.56	4
Our study	97.78	4

a IQ-OTH/NCCD: Iraq-Oncology Teaching Hospital/National Center for Cancer Diseases.

b CT: computed tomography.

## Discussion

### Principal Findings

The experimental results of the study demonstrated that the RPS DA technique significantly enhanced the diagnostic performance of both CNN and transformer architectures for lung cancer diagnosis from CT scan images. Our comprehensive evaluation demonstrated that RPS consistently outperformed 4 established augmentation methods (CutMix, Random Erasing, MixUp, and Cutout) across multiple performance metrics and diverse experimental conditions. The superior efficacy of RPS stems from its unique capacity to preserve critical anatomical content while generating clinically meaningful variations through controlled intraimage pixel swapping. This characteristic makes RPS particularly valuable for medical imaging applications where maintaining content integrity is essential for an accurate diagnosis.

For CNN architectures, specifically ResNet-34, RPS yielded remarkable performance improvements. ResNet-34 achieved peak accuracies of 97.56% for the IQ-OTH/NCCD dataset and 97.78% for the chest CT scan images dataset, with corresponding AUROC scores of 98.61% and 99.46%, respectively, at 512×512 image resolution. The technique’s effectiveness with MobileNetV3 (96.65% accuracy and 98.0% AUROC for the IQ-OTH/NCCD dataset; 96.83% accuracy and 98.75% AUROC for the chest CT scan images dataset) is particularly notable given this model’s lightweight architecture, suggesting RPS’s potential for deployment in resource-constrained clinical settings where efficient models are often preferred [56]. The study results represent a substantial advancement over conventional augmentation approaches, as RPS effectively addresses the inherent limitation of CNNs in capturing global relationships by creating localized variations that enhance feature learning while preserving diagnostically relevant image features.

The transformer-based architectures (Vision Transformer and Swin Transformer) showed particularly notable improvements when augmented with RPS. While transformer models conventionally demand large-scale training datasets to achieve peak performance, RPS effectively compensated for data limitations by generating variations that preserved the overall image content for proper attention mechanism functioning. For the Vision Transformer, RPS augmentation significantly enhanced performance, reaching 92.38% accuracy and 96.93% AUROC on the IQ-OTH/NCCD dataset and 86.67% accuracy and 96.32% AUROC on the chest CT scan images dataset. The Swin Transformer demonstrated robust performance gains, achieving 94.82% accuracy and 98.12% AUROC on the IQ-OTH/NCCD dataset and 96.19% accuracy and 98.90% AUROC on the chest CT scan images dataset when enhanced with RPS. The study results showed that RPS enables transformer models to develop more robust and clinically relevant feature representations, even with limited training data.

Our comparative analysis revealed RPS’s consistent dominance across evaluation metrics and experimental conditions. While CutMix showed marginal advantages in specific scenarios (notably a 0.31% accuracy improvement with ResNet-34 at 512×512 image resolution), RPS maintained substantially better AUROC scores (5.31% higher in the same comparison), indicating more reliable diagnostic discrimination capability. This performance pattern held true across both the IQ-OTH/NCCD and chest CT scan images datasets, with RPS consistently ranking the highest in our comprehensive evaluation framework. Importantly, while conventional augmentation techniques sometimes degraded model performance in certain scenarios [38,40], RPS demonstrated universal performance enhancement across all tested conditions. Three fundamental characteristics explain RPS’s exceptional effectiveness. The first characteristic is anatomical content preservation. Unlike methods that erase or mix image regions, RPS maintains all original diagnostic information while creating realistic variations through a controlled, systematic, random patch-based pixel swap within carefully defined ROIs. This approach preserves the clinical relevance of training samples while providing valuable data diversity. The second characteristic is architecture agnostic adaptability. The technique’s parameter-free implementation and tunable swap area factor enable optimal performance across diverse model architectures without requiring architecture-specific adjustments. This flexibility makes RPS particularly valuable for medical imaging research, where multiple architectures may be explored. The third characteristic is clinical pathological relevance. By restricting pixel swaps to anatomically plausible regions within lung tissue (especially when combined with ROI segmentation), RPS enhances the learning of pathological features that may appear anywhere in the pulmonary anatomy, a crucial capability given the unpredictable spatial distribution of malignant nodules in many cancer cases [61].

Validation experiments using independently acquired datasets with different scanning protocols and equipment configurations demonstrated RPS’s superior generalization capabilities. The technique achieved these results while adding minimal computational overhead (statistically insignificant increases in training time, *P*>.05), making it practical for real-world clinical implementation. Furthermore, RPS showed excellent compatibility with conventional augmentation methods, providing additional performance gains when combined with rotation and flipping operations, which suggests easy integration into existing medical image processing pipelines.

These findings offer significant implications for the development of computer-aided diagnosis systems. RPS directly addresses two fundamental challenges in medical AI: (1) the scarcity of annotated medical imaging data and (2) the limited generalizability of many models across different clinical settings [23]. By consistently outperforming current state-of-the-art techniques while maintaining computational efficiency, RPS emerges as a versatile solution suitable for both research investigations and clinical deployment. Additionally, the technique’s effectiveness suggests promising applications in educational settings for training radiologists, where realistic image variations could enhance learning without requiring additional patient scans.

### Conclusions

The findings of this study demonstrate that RPS is a robust and versatile DA technique that significantly enhances the performance of both CNN and transformer architectures for lung cancer diagnosis from CT scan images. By preserving anatomical content while introducing meaningful variability, RPS outperforms existing augmentation methods across multiple metrics and datasets, achieving improved accuracy and AUROC scores. Its computational efficiency, adaptability to diverse architectures, and ability to improve generalization make it particularly valuable for medical imaging applications where data scarcity and model reliability are critical challenges. RPS not only advances the technical frontier of DA but also holds immediate promise for improving computer-aided diagnosis systems in clinical practice. Future work will explore its extension to other medical imaging modalities (magnetic resonance, ultrasound, and x-ray imaging) and extension to 3D applications.

## Abbreviations

AUROC: area under the receiver operating characteristic curve
CNN: convolutional neural network
CT: computed tomography
DA: data augmentation
DL: deep learning
IQ-OTH/NCCD: Iraq-Oncology Teaching Hospital/National Center for Cancer Diseases
MVSF: multivalue swap area factor
ROI: region of interest
RPS: Random Pixel Swap
SVSF: single-value swap area factor
